# Functional interplay between protein arginine methyltransferases in *Trypanosoma brucei*

**DOI:** 10.1002/mbo3.191

**Published:** 2014-07-07

**Authors:** Kaylen Lott, Lu Zhu, John C Fisk, Danielle L Tomasello, Laurie K Read

**Affiliations:** 1 Department of Microbiology and Immunology, University at Buffalo School of MedicineBuffalo, New York, 14214

**Keywords:** Arginine methylation, posttranslational modifications, PRMTs, Trypanosomes

## Abstract

Arginine methylation is a common posttranslational modification that has far-reaching cellular effects. *Trypanosoma brucei* is an early-branching eukaryote with four characterized protein arginine methyltransferases (PRMTs), one additional putative PRMT, and over 800 arginine methylated proteins, suggesting that arginine methylation has widespread impacts in this organism. While much is known about the activities of individual *T. brucei* PRMTs (TbPRMTs), little is known regarding how TbPRMTs function together in vivo. In this study, we analyzed single and selected double TbPRMT knockdowns for the impact on expression of TbPRMTs and global methylation status. Repression of TbPRMT1 caused a decrease in asymmetric dimethylarginine and a marked increase in monomethylarginine that was catalyzed by TbPRMT7, suggesting that TbPRMT1 and TbPRMT7 can compete for the same substrate. We also observed an unexpected and strong interdependence between TbPRMT1 and TbPRMT3 protein levels. This finding, together with the observation of similar methyl landscape profiles in TbPRMT1 and TbPRMT3 repressed cells, strongly suggests that these two enzymes form a functional complex. We show that corepression of TbPRMT6/7 synergistically impacts growth of procyclic-form *T. brucei*. Our findings also implicate the actions of noncanonical, and as yet unidentified, PRMTs in *T. brucei*. Together, our studies indicate that TbPRMTs display a functional interplay at multiple levels.

## Introduction

Arginine methylation is a common posttranslational modification entailing the transfer of a methyl group from the methyl donor *S*-adenosylmethionine (AdoMet) to the terminal nitrogen of a peptidyl arginine. Methylarginines are most commonly found in RGG, RG, or RXR motifs, although this does not always hold true (Fisk et al. 2013; Lott et al. 2013). Arginine methylation affects myriad cellular functions including transcriptional regulation, mRNA splicing, DNA repair, and signal transduction (Bedford and Clarke 2009). Although the addition of a methyl group does not affect the charge of the arginine residue, it does increase its mass which can have both positive and negative effects on its hydrogen bonding capabilities (Horowitz and Trievel 2012). Numerous studies have shown that arginine methylation can influence both protein–protein and protein–nucleic acid interactions either positively or negatively (Bedford 2007; Bedford and Clarke 2009).

The parasitic protozoan, *Trypanosoma brucei,* is the causative agent of African sleeping sickness in humans and nagana in African livestock. Unlike most eukaryotes, *T. brucei* regulates gene expression primarily posttranscriptionally, relying on processes such as RNA stabilization, translation, and editing (Clayton and Shapira 2007; Kramer 2012). Thus, RNA-binding proteins are key regulators of parasite function. RNA-binding proteins constitute a large number of arginine methylated proteins in higher organisms (Pahlich et al. 2006; Bedford and Clarke 2009), and this prompted us to examine the arginine methylome of *T. brucei* to ask if the same is true in this early-branching eukaryote. Indeed, in a global proteomics screen in *T. brucei*, we identified 136 RNA-binding and metabolic proteins harboring arginine methylmarks (Fisk et al. 2013; Lott et al. 2013). In total, 844 arginine methylated proteins were detected, accounting for approximately one tenth of all proteins in *T. brucei*, suggesting a widespread impact of this modification on parasite biology.

Arginine methylation is catalyzed by a family of enzymes termed protein arginine methyltransferases (PRMTs). PRMTs are divided into three major types based on the end product they catalyze, with all types being able to catalyze *ω*-*N*^*G*^-monomethylarginine (MMA). Type I PRMTs catalyze asymmetric *ω*-*N*^*G*^,*N*^*G*^-dimethylarginine (ADMA) as a final product, while type II PRMTs produce symmetric *ω*-*N*^*G*^,*N*^*G*^-dimethylarginine (SDMA). Type III PRMTs catalyze solely MMA. We have characterized four of the five putative PRMTs encoded in the *T. brucei* genome in terms of their enzymatic activities and the effects of RNA interference (RNAi)-mediated repression on trypanosome growth (Pelletier et al. 2005; Pasternack et al. 2007; Fisk et al. 2009, 2010; Fisk and Read 2011). TbPRMT1 displays type I activity, and based on a global reduction in in vivo–labeled ADMA upon its depletion, is considered to be the major type I PRMT in *T. brucei* (Pelletier et al. 2005). In vitro, TbPRMT1 activity is weak, but it has been shown to methylate proteins with RG motifs (Pelletier et al. 2005; Goulah et al. 2006). The other characterized type I PRMT, TbPRMT6, is the only TbPRMT shown by RNAi to be essential for optimal parasite growth and cytokinesis (Fisk et al. 2010). Although TbPRMT6 has a very narrow substrate range in vitro, it presumably methylates one or more substrates in vivo that are critical for parasite growth (Fisk et al. 2010). TbPRMT5 is the only type II PRMT characterized in *T. brucei*, and it methylates a relatively broad range of substrates in vitro. In *T. brucei*, immunoprecipitated TbPRMT5 associated with three possible substrates, two of which (Tb10.61.2130 and Tb09.160.1400) were later identified as containing methylarginine (Pasternack et al. 2007; Lott et al. 2013). TbPRMT7 was one of the first *bone fide* type III PRMTs to be characterized in any organism. In vitro*,* TbPRMT7 is extraordinarily active and promiscuous with regard to substrate specificity (Fisk et al. 2009). Finally, TbPRMT3 is a putative type I enzyme most homologous to human PRMT3. However, TbPRMT3 lacks key catalytic residues in the Thr-His-Trp (THW) loop that are typically present in type I enzymes. It also contains an E to D substitution in the double E loop that is critical to the activities of all PRMTs (Fisk and Read 2011). We have been unable to assess the activity of TbPRMT3, as the recombinant protein is inactive to date. TbPRMTs localize primarily to the cytoplasm, with no evidence of mitochondrial localization (Pasternack et al. 2007; Fisk et al. 2010). Nevertheless, we identified over 150 methylated proteins in the mitochondria of *T. brucei* (Fisk et al. 2013), suggesting that there remain uncharacterized TbPRMTs.

Little is known regarding the functional interactions between TbPRMTs, or if suppressing one TbPRMT affects the activity of others. PRMTs have been reported to act redundantly, but with most of the studies coming from the epigenetics field, the prevalence of this interplay is far from understood (Di Lorenzo and Bedford 2011). Moreover, in mammals, it was shown that loss of PRMT1 led to a 50% reduction in ADMA, and a dramatic increase in MMA and SDMA (Dhar et al. 2013). This study provided the first global evidence showing that unmasked arginines can serve as substrates for multiple PRMTs. Interestingly, this phenomenon was observed only upon depletion of PRMT1, suggesting that regulatory measures might be in place to modulate PRMT1 activity and permit access of other PRMTs to specific arginine residues. In this study, we sought to determine whether TbPRMTs can compensate for one another, and define how the methyl landscape changes upon depletion of specific TbPRMTs. In agreement with studies in human cells on PRMT1, we show that depletion of TbPRMT1 causes a marked decrease in ADMA, concomitant with a dramatic increase in MMA marks. We further go on to characterize these new MMA marks as TbPRMT7-catalyzed products. Unlike what is observed in mammalian cells, however, we show that repression of TbPRMT3 leads to a similar phenotype as TbPRMT1 repression. We also made the surprising finding that TbPRMT1 and TbPRMT3 are interdependent with regard to their stability, suggesting that these enzymes form a functional hetero-oligomer. Finally, we demonstrate a synergistic effect of TbPRMT6/7 repression of procyclic-form (PF) growth, and provide additional evidence for the existence of noncanonical PRMTs in *T. brucei*. These studies begin to unveil the complex nature of protein arginine methylation, evident even in one of the earliest-branching eukaryotes.

## Materials and Methods

### *Trypanosoma brucei* cell culture and generation of knockdown cell lines

PF *T. brucei* strain 29-13, containing a Tet-inducible system and viral T7 RNA polymerase (Wirtz et al. 1999), and all knockdown lines derived from this strain were grown in SM media supplemented with 10% fetal bovine serum (Cunningham 1977). RNA interference lines that target TbPRMT6 or TbPRMT7 open reading frames were previously described (Fisk et al. 2009, 2010) and were derived again using the same construct for this study. To create cells that express tetracycline-inducible dsRNA-targeting TbPRMT3 for RNAi, the full-length TbPRMT3 open reading frame was excised from pGEX4T-1-TbPRMT3 (see below) using BamHI and XhoI and ligated into the BamHI-XhoI sites of RNAi vector p2T7-177 (Wirtz et al. 1999) creating p2T7-177-TbPRMT3. For creation of an RNAi line-targeting TbPRMT1 using p2T7-177 (as opposed to the previously described pZM stem loop vector in (Pelletier et al. 2005)), the full ORF of TbPRMT1 was amplified using PRMT1 5′ BamHI (5′-GAGGATCCATGACGGTGGACGCAAATGCCGCC-3′) and PRMT1 3′ XhoI (5′-GGCTCGAGCTACCGCAGCCGAAAATCCTGGTC-3′) and established in pJET (CloneJet cloning kit; Fermentas, Pittsburgh PA, USA). Using BamHI and XhoI excision, the 1054-bp TbPRMT1 ORF was cloned into p2T7-177, yielding p2T7-177-TbPRMT1. To produce cell lines that target multiple PRMT genes, the following strategies were used. An RNAi construct that targets both ORFs of TbPRMT6 and TbPRMT7 was created by first amplifying a 676-bp fragment of TbPRMT7 using PRMT7 5′XbaI (5′-GGTCTAGATCGGAGATATTTGGAACG-3′) and PRMT7 3′ XbaI (5′-GATCTAGAGGTTGTTTTGCCCTCGCACTCAGTC-3′). This fragment was first established in the pJET cloning vector, excised using XbaI, followed by ligation into the two XbaI sites of p2T7-177. Next, a fragment of TbPRMT6 was amplified using PRMT6 5′ ClaI (5′-GTATCGATTGGCGTAGCAGTGCTTC-3′) and PRMT6 3′ HindIII (5′-GGAAGCTTTTTTAACTCGAGCTCAATGGTG-3′). This TbPRMT6 fragment was established in the pJET cloning vector, and a 702-bp fragment was subsequently excised using ClaI and an endogenous XhoI site in the C-terminus of TbPRMT6. This fragment was finally ligated into the ClaI-XhoI sites of p2T7-177-TbPRMT7, yielding p2T7-177-PRMT6-PRMT7. To produce a RNAi line simultaneously targeting the 3′untranslated region (UTRs) of TbPRMT1 and TbPRMT7, a 364-bp fragment of the TbPRMT7 3′UTR was PCR amplified using TbPRMT7 UTR 5′ BamHI (5′-GAGGATCCTGGAGGAAGGGCGTGGC-3′) and TbPRMT7 UTR 3′ XhoI (5′-GACTCGAGTGGGAGAGTGAAGAAAG-3′). After establishment of a pJET intermediate, the 3′UTR fragment was liberated using BamHI and XhoI, followed by ligation into the BamHI-XhoI sites of p2T7-177, yielding p2T7-177-TbPRMT7 3′UTR. Next, a 401 bp of the PRMT1 3′UTR was PCR amplified using TbPRMT1 3′ UTR 5′ XbaI (5′-GGTCTAGAGAAGGTTGTGAAGAGTTATCACG-3′) and TbPRMT1 3′ UTR 3′ BamHI (5′-GAGGATCCGAATCCTCGGTGCCGATCACGC-3′)and ligated into pJET. The TbPRMT1 3′UTR fragment was then ligated into the XbaI-BamHI sites of p2T7-177-TbPRMT7 3′UTR to produce p2T7-177-TbPRMT1-TbPRMT7 3′UTRs. NotI-linearized RNAi vectors were transfected into *T. brucei* 29-13 cells, and cells harboring these constructs were selected using 2.5 *μ*g/mL phleomycin. Cells were induced with 4 *μ*g/mL of tetracycline and harvested at days 3 and 4 postinduction for protein collection, or at day 3 postinduction for RNA isolation. For growth curves, cells at a starting concentration of 8.0 × 10^5^ cell/mL were induced with 4 *μ*g/mL of tetracycline and diluted as necessary every 48 h. Values from three independent growth experiments were averaged to generate growth curves with experimental error bars depicting SD.

### Antibodies

ASYM24 was purchased from EMD Millipore and used at a dilution of 1:1000. MMA(R*GG) and MMA(MeR^4^) were purchased from Cell Signaling (Danvers, MA, USA), and both were used at a dilution of 1:1000. TbPRMT1, TbPRMT6, and TbPRMT7 antibodies were described in Fisk et al., 2010. P22 and TbRGG2 load control antibodies were described in Hayman et al., 2001, and Fisk et al., 2008, respectively. Antibodies against the TbPRMT3 peptide IETKGTYNYQRY were raised in rabbits (Bethyl Laboratories, Montgomery, TX, USA). These antibodies were further affinity purified using recombinant GST-TbPRMT3 that was generated as follows. The TbPRMT3 open reading frame (Tb927.10.3560) was PCR amplified from oligo(dT)-primed cDNA extracted from PF *T. brucei* (29-13) RNA using the primers PRMT3-5′-BamHI (5′-GAGGATCCATGTCACCAAAGAAAAACTCGGC-3′) and PRMT3-3′-XhoI (5′-GGCTCGAGATACCTTTGGTAGTTGTACGTGC-3′). The resultant product was cloned into pJET. TbPRMT3 was excised from pJET-TbPRMT3 and ligated into the BamHI and XhoI sites of pGEX4T-1 (GE Healthcare Life Sciences, Pittsburgh, PA, USA). The resultant plasmids were then transformed into Rosetta strain *Escherichia coli* cells (Novagen, Darmstadt, Germany) for expression. GST-tagged TbPRMT3 was purified using single-step glutathione-agarose (Invitrogen, Grand Island, NY, USA). For affinity purification of anti-TbPRMT3 antibodies, GST-TbPRMT3 was separated by sodium dodecyl sulfate polyacrylamide gel electrophoresis (SDS-PAGE) and immobilized on nitrocellulose membrane. GST-TbPRMT3 was stained using Ponceau stain, excised as membrane chips and bound in batch with anti-TbPRMT3 peptide antibodies. The antibodies were washed using phosphate buffered saline (PBS) with 0.1% Tween-20, and then eluted with 100 mmol/L glycine (pH 2.5). Resultant elutants were neutralized with 1 mol/L Tris (pH 8.0).

### Western blots

Proteins were separated by 12.5% SDS-PAGE and transferred to PVDF membrane. Antibodies used are described above. Blots were visualized using Bio-Rad (Hercules, CA, USA) ChemiDoc imager. Quantification of TbPRMT levels was performed using Bio-Rad Image Lab software and normalized against load controls.

### qRT-PCR analysis

Total RNA was extracted from 1 × 10^8^ cells. Four micrograms of RNA was treated with a DNase kit (Ambion, Grand Island, NY, USA) to remove any residual DNA. RNA was reverse transcribed using random hexamers (Applied Biosystems, Grand Island, NY, USA). The following primer pairs specific for each TbPRMT were utilized: primers complementary to the 5′ end of TbPRMT1 ORF (5′-TATGTTCATGCGCTTTCTGT-3′) and to the 3′ end of TbPRMT1 ORF(5′-GAGGGGAATATGGAGTTGTG-3′); primers complementary to the 5′ end of TbPRMT1 3′UTR (5′-CGTTCTCACTGCTTTGTTTG-3′) and to the 3′ end of TbPRMT1 3′UTR (5′-TTTCCGAAGAAGTGGAAGAG-3′); primers complementary to the 5′ end of TbPRMT3 3′UTR (5′-GCGTGTATGGGGGTATTTAG-3′) and to the 3′ end of TbPRMT3 3′UTR (5′-AACATGTTCTTGCACACGAC-3′); and primers complementary to the 5′ end of TbPRMT6 ORF (5′-GGTGTGGAGATGCATATTAGTG-3′) and to the 3′ end of TbPRMT6 ORF (5′-CACGCATTTGTAAGCAAAAC-3′); and primers complementary to the 5′ end of TbPRMT7 ORF (5′-GCGTGATTCTTCACATGC-3′) and to the 3′ end of TbPRMT7 ORF (5′-CACACTGTTCACCCTCATTTC-3′).

## Results

### TbPRMT1 depletion leads to TbPRMT3 destabilization

**Figure 1:**
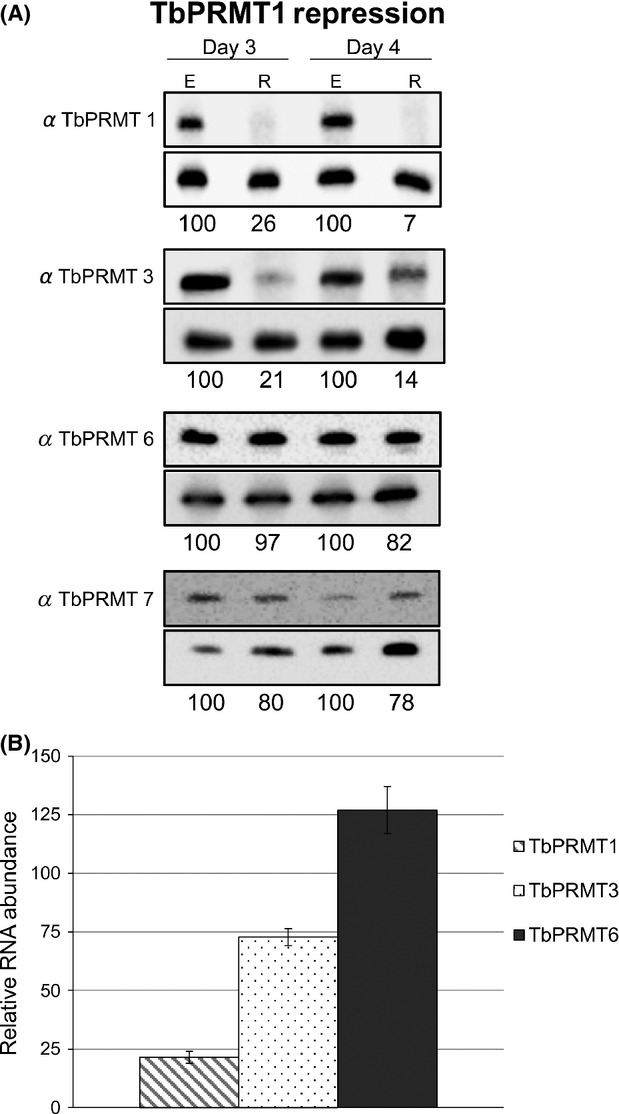
TbPRMT expression patterns upon TbPRMT1 repression. (A) *Trypanosoma brucei* procyclic-form cells either expressing (E) or repressed (R) for TbPRMT1 were harvested on day 3 or 4 postinduction, lysed in SDS-PAGE sample buffer, and resolved via 12.5% SDS-PAGE. Each lane contains 5 × 10^6^ cell equivalents. Western blot analysis was carried out using antibodies specific to each PRMT. Load controls shown under each blot. The protein p22 was used as a load control in all western blots except for the TbPRMT7 blot, in which the mitochondrial protein, TbRGG2 was used. Quantification of TbPRMTs is represented as normalized percentages compared to TbPRMT1-expressing cells. (B) Quantitative RT-PCR analysis of TbPRMT1, TbPRMT3, and TbPRMT6 RNA levels in TbPRMT1-repressed cells relative to those in TbPRMT1-expressing cells. RNA levels were normalized against 18S rRNA and represent the average and SD of 3–6 determinations.

TbPRMT1 is the major type 1 PRMT in *T. brucei* as demonstrated by a dramatic decrease in ADMA upon in vivo labeling of TbPRMT1 knockdown cells (Pelletier et al. 2005). TbPRMT6 is also a type I enzyme, and TbPRMT3 is predicted to have type I activity based on its homology to human PRMT3, although its activity has not been biochemically characterized as the recombinant enzyme is inactive (Fisk et al. 2010; Fisk and Read 2011). We first wanted to ask whether PF *T. brucei* attempt to compensate for depletion of TbPRMT1 by increasing either of the other two type I PRMTs or the type III TbPRMT7, as was observed at later time points following PRMT1 removal in human cells (Dhar et al. 2013). To this end, we repressed expression of TbPRMT1 by induction of RNAi with tetracycline, and we harvested cells at 3 and 4 days postinduction. We assessed the abundances of TbPRMTs by western blot using antibodies specific to each enzyme (Fisk et al. 2010). As expected, TbPRMT1 protein levels are depleted to 26% and 7% of wild type on days 3 and 4, respectively (Fig.1A). Surprisingly, TbPRMT3 was also depleted, and quantification indicated that TbPRMT3 protein levels are reduced to a similar extent as TbPRMT1 levels. In contrast, the abundance of TbPRMT6 and the type III enzyme TbPRMT7 were nearly unchanged when TbPRMT1 was repressed. To ensure that the dramatic TbPRMT3 depletion that we observed upon TbPRMT1 repression was due to regulation at the protein level rather than off-target effects of the RNAi, we assessed RNA levels in TbPRMT1 knockdown cells. Total RNA was harvested from cells on day 3 postinduction and quantitative reverse-transcription polymerase chain reaction (qRT-PCR) analysis was performed using primers specific for TbPRMT1, TbPRMT3, or TbPRMT6. TbPRMT1 RNA was depleted to 22% that of wild type, in line with the level of protein depletion. TbPRMT3 RNA was depleted to 73% that of wild-type levels. TbPRMT6 RNA was increased to 127% of wild-type levels although protein levels were unchanged, suggesting that the modest decrease in TbPRMT3 RNA is within the range of experimental error. Thus, we conclude that the observed decrease in TbPRMT3 takes place at the protein level (see also Fig. 3). Collectively, these data indicate that neither type I nor type III PRMTs undergo compensatory increases when TbPRMT1 is repressed. On the contrary, the TbPRMT3 protein is concomitantly depleted upon TbPRMT1 knockdown.

### TbPRMT1 repression unmasks substrates for TbPRMT7

**Figure 2:**
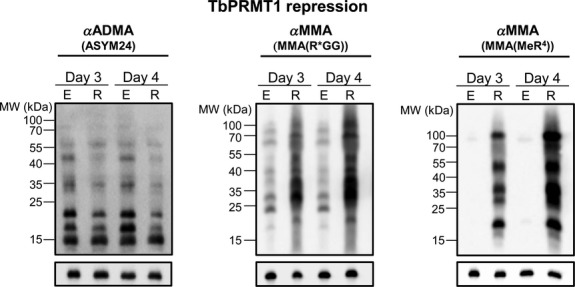
TbPRMT1 repression leads to a decrease in ADMA and an increase in MMA. *Trypanosoma brucei* procyclic-form cells either expressing (E) or repressed (R) for TbPRMT1 were lysed in SDS-PAGE sample buffer and resolved via 12.5% SDS-PAGE. Each lane contains 1 × 10^7^ cell equivalents. Western blot analysis was carried out using three different antimethylarginine antibodies: ASYM24 detects ADMA; MMA(R*GG) and MMA(MeR^4^) detect MMA. p22 (bottom) is shown as a load control.

In mammalian cells, PRMT1 depletion caused marked changes in substrate methylation status, thereby prompting us to ask if repression of TbPRMT1 (and coincident TbPRMT3 depletion) would dramatically alter the methyl landscape in *T. brucei*. In particular, we asked whether TbPRMT1 repression leads to a substantial increase in MMA, as observed in mammalian cells (Dhar et al. 2013). Using the TbPRMT1 repressed cells described above, total protein was isolated, separated by SDS-PAGE, and probed with antibodies specific for ADMA and MMA. Two anti-MMA antibodies were used, each recognizing MMA, although in different contexts. MMA(Me-R^4^) recognizes MMAs with little to no sequence specificity for the surrounding residues while the MMA(R*GG) antibody prefers MMAs in the context of RGG motifs (Dhar et al. 2013). Consistent with the fact that TbPRMT1 is the major type I PRMT, depletion of TbPRMT1 caused a significant decrease in ADMA levels on numerous proteins (Fig.2, left). The remaining ADMA signal may be due to the activities of other type I PRMTs in the cell or the small fraction of TbPRMT1 remaining after RNAi. It is important to note that substrates detected by ASYM24 offer a limited view of the total cellular arginine methylome due to the antibody's context bias (Fisk et al. 2013; Lott et al. 2013). TbPRMT1-expressing cells contain numerous proteins that are recognized by both the MMA(R*GG) and MMA(MeR^4^) antibodies, with different patterns seen due to the different specificities (Fig.2, middle and right). When TbPRMT1 was repressed, the level of MMA was dramatically increased. MMA increases were observed in the context of both RGG motifs as recognized by the MMA (R*GG) antibody and in the absence of motif specificity as recognized by the MMA (MeR^4^) antibody. Several MMA-containing proteins that were detected in wild-type cells exhibited increased MMA labeling when TbPRMT1 was repressed, and several new MMA-containing proteins became apparent.

**Figure 3:**
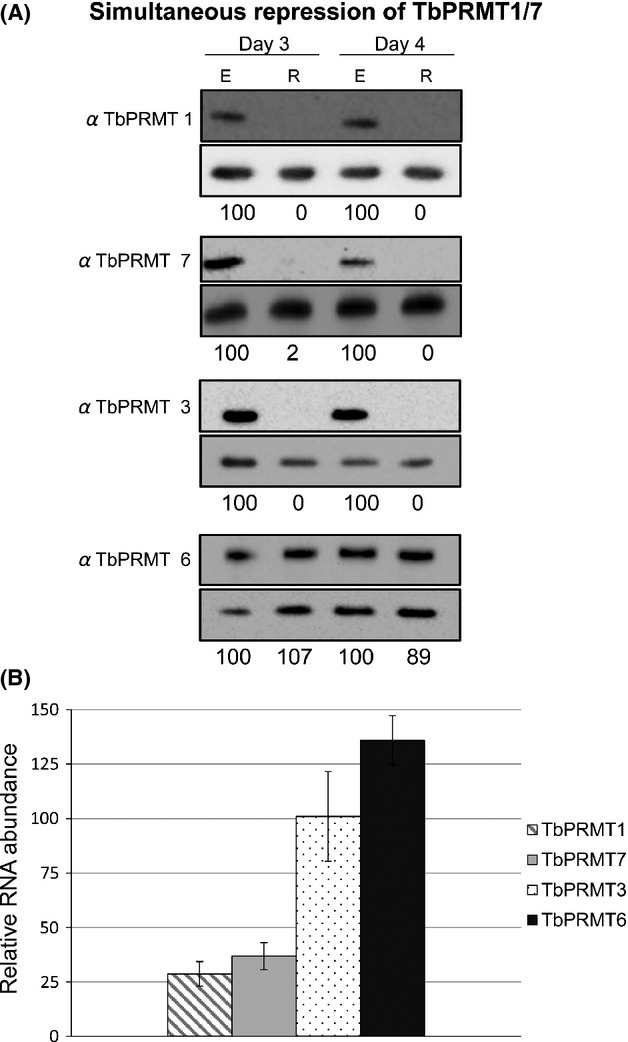
Expression patterns of TbPRMTs upon simultaneous TbPRMT1/7 knockdown. (A) *Trypanosoma brucei* procyclic-form cells either expressing (E) or repressed (R) for TbPRMT1/7 were lysed in SDS-PAGE sample buffer and resolved via 12.5% SDS-PAGE. Each lane contains 5 × 10^6^ cell equivalents. Western blot analysis was carried out using antibodies specific to each PRMT. Load controls shown under each blot. The protein p22 was used as a load control in all western blots except for the TbPRMT7 blot, in which the mitochondrial protein, TbRGG2, was used. Quantification of TbPRMTs is represented as normalized percentages compared to TbPRMT1/7-expressing cells. (B) Quantitative RT-PCR analysis of TbPRMT1, TbPRMT7, TbPRMT3, and TbPRMT6 RNA levels in TbPRMT1/7-repressed cells relative to those in TbPRMT1/7-expressing cells. RNA levels were normalized against 18S rRNA and represent the average and SD of 3–6 determinations.

We considered the possible mechanisms by which TbPRMT1 repression could lead to an increase in the MMA content of proteins. MMA is an intermediate in the synthesis of ADMA by TbPRMT1. Because TbPRMT1 is only decreased and not abolished, it is possible that the less abundant TbPRMT1 that remains after RNAi simply acts in a more distributive fashion, thereby leading to a decrease in the final product, ADMA, and an increase in the MMA intermediate. Alternatively, the decrease in TbPRMT1-catalyzed methylmarks may unmask substrates for the type III TbPRMT7, which synthesizes MMA as a final product. To distinguish between these models, we generated a PF *T. brucei* cell line in which both TbPRMT1 and TbPRMT7 were repressed via inducible RNAi. To ensure that the remaining TbPRMTs were not compensating for the loss of both TbPRMT1 and TbPRMT7, we again assayed the levels of different PRMTs. As shown in Figure3, TbPRMT1 and TbPRMT7 were knocked down to levels undetectable by western blot analysis. In agreement with the single TbPRMT1 knockdown, TbPRMT3 protein levels were again significantly reduced compared to wild-type cells. We verified that this depletion was at the protein level, and that TbPRMT3 RNA levels were not affected due to off-target effects of the RNAi (Fig.3B). qRT-PCR analysis shows that TbPRMT1 RNA levels were depleted to the same degree in the TbPRMT1/7 knockdown cells as in the single TbPRMT1 knockdowns (Fig.1B), and that TbPRMT7 RNA was reduced to 35% of wild-type levels. Similar to the TbPRMT1 knockdown cells, TbPRMT6 levels were not significantly changed in TbPRMT1/7 knockdown cells (Fig.3). Simultaneous TbPRMT1/7 knockdown resulted in a very slight, but reproducible, growth defect (data not shown) that was not observed when either enzyme was knocked down separately (Pelletier et al. 2005; Fisk et al. 2009).

**Figure 4:**
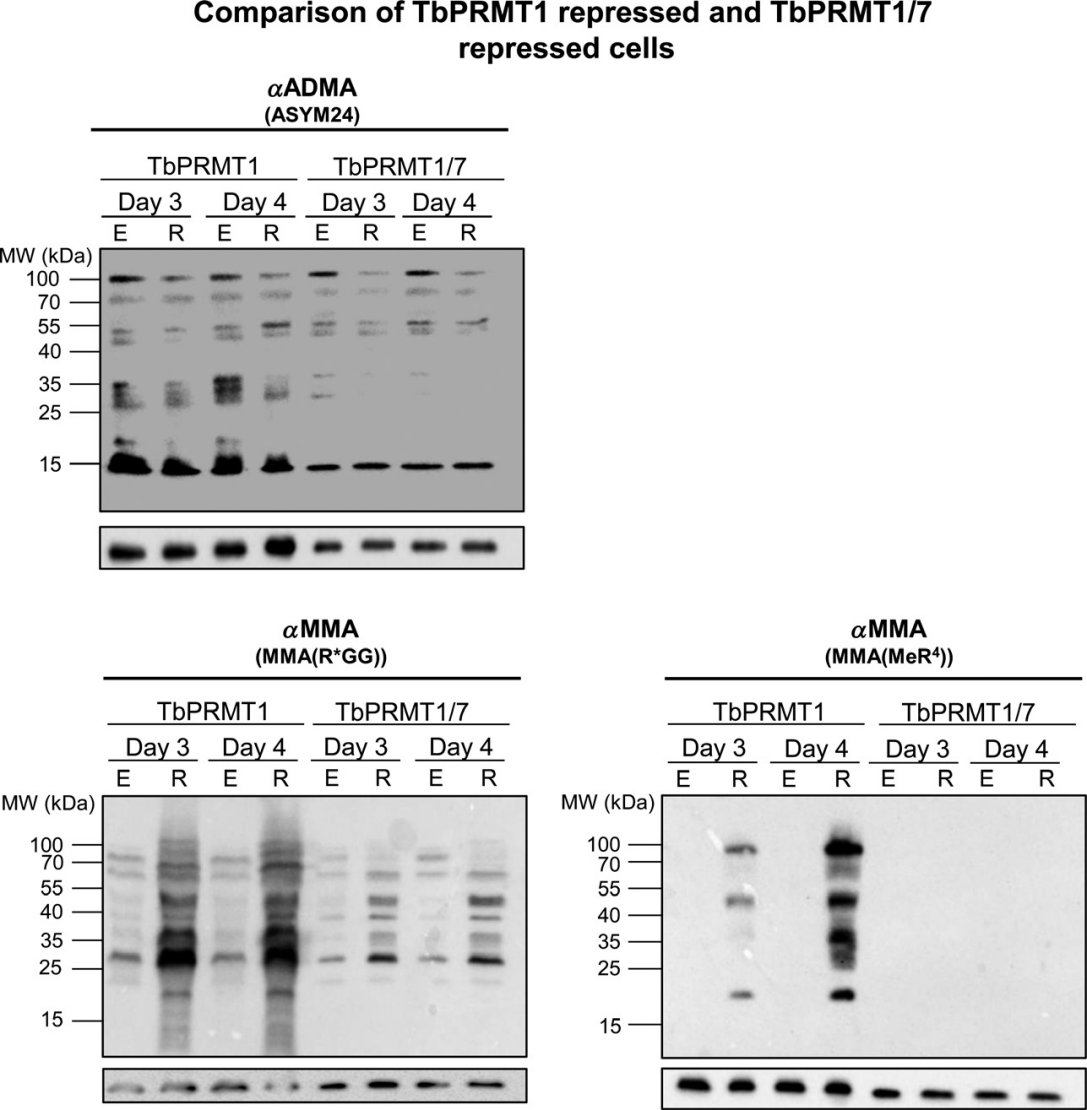
Simultaneous TbPRMT1/7 repression reveals substrate scavenging by TbPRMT7. *Trypanosoma brucei* procyclic-form cells either expressing (E) or repressed (R) for TbPRMT1, or both TbPRMT1 and TbPRMT7 (TbPRMT1/7), were lysed in SDS-PAGE sample buffer and resolved via 12.5% SDS-PAGE. Each lane contains 1 × 10^7^ cell equivalents. Western blot analysis was carried out using three different antimethylarginine antibodies: ASYM24 detects ADMA, and MMA(R*GG) and MMA(MeR^4^) detect MMA. The p22 load control is shown under each blot.

Having shown that expression of both TbPRMT1 and TbPRMT7 proteins is repressed in the double knockdown line, we then verified that ADMA levels are decreased as expected due to TbPRMT1 repression (Fig.4, top). As with the single TbPRMT1 knockdown (Fig.2), ADMA decreases were more apparent on some proteins than on others. Next, to determine whether the dramatic increase in MMA upon TbPRMT1 knockdown is due to unmasking of TbPRMT7 substrates, we compared the MMA signals in TbPRMT1 knockdowns to those in TbPRMT1/7 double knockdowns (Fig.4, bottom). If TbPRMT7 is scavenging substrates that are unmasked by TbPRMT1 repression, then we would expect that simultaneous TbPRMT1/7 repression would substantially diminish the MMA signal that occurs upon TbPRMT1 knockdown. Indeed, MMA levels in the TbPRMT1/7 double knockdown were drastically reduced compared to those observed upon knockdown of TbPRMT1 alone (Fig.4, bottom). MMA detected with the MMA(R*GG) antibody was still slightly elevated in TbPRMT1/7 knockdown cells compared to TbPRMT1/7-expressing cells, although not nearly to the same extent as in the TbPRMT1 knockdown alone. The increased MMA observed in the MMA(MeR^4^) context in the TbPRMT1 knockdown was abolished by simultaneous TbPRMT1/7 knockdown. These data indicate that the increase in MMA observed upon TbPRMT1 repression is primarily due to TbPRMT7 scavenging now unmasked substrates.

### TbPRMT3 repression reveals interdependence between TbPRMT3 and TbPRMT1

**Figure 5:**
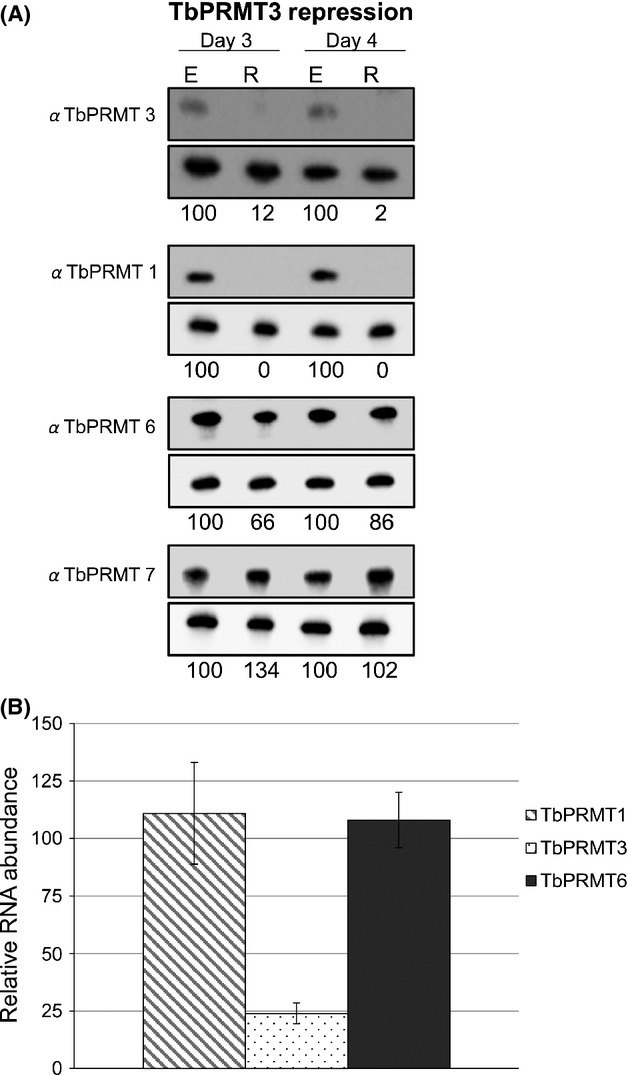
TbPRMT3 repression leads to a decrease in TbPRMT1 protein levels. (A) *Trypanosoma brucei* procyclic-form cells either expressing (E) or repressed (R) for TbPRMT3 were lysed in SDS-PAGE sample buffer and resolved via 12.5% SDS-PAGE. Each lane contains 5 × 10^6^ cell equivalents. Western blot analysis was carried out using antibodies specific to each TbPRMT. p22 serves as a load control and is shown below each respective TbPRMT western blot. Quantification of TbPRMTs is represented as normalized percentages compared to TbPRMT3-expressing cells. (B) Quantitative RT-PCR analysis of TbPRMT1, TbPRMT3, and TbPRMT6 RNAs in TbPRMT3-repressed cells relative to those in TbPRMT3-expressing cells. RNA levels were normalized against 18S rRNA and represent the average and SD of 3–6 determinations.

TbPRMT3 is a putative type I PRMT. When compared to human PRMTs, it displays the highest sequence homology to PRMT3, although TbPRMT3 lacks conserved residues in the THW loop and harbors an E to D substitution in the double E loop (Frankel and Clarke 2000; Fisk and Read 2011). To date, we have not been successful in obtaining enzymatically active recombinant TbPRMT3. We reasoned that repression of the TbPRMT3 in vivo followed by western blot analysis might provide insight into its activity. To this end, we established a PF *T. brucei* cell line containing a tetracycline-regulated TbPRMT3 RNAi construct. Upon tetracycline addition for 3 or 4 days, TbPRMT3 was repressed to 12% and 2% of levels in TbPRMT3-expressing cells (Fig.5A). No growth defect was observed under these conditions. We next sought to determine whether TbPRMT3 repression affects the expression patterns of other TbPRMTs. At days 3 and 4 postinduction we did not observe any major changes in the protein levels of TbPRMT6 or TbPRMT7. In striking contrast, TbPRMT1 expression was essentially abolished upon TbPRMT3 repression according to western blot analysis. qRT-PCR analysis confirmed that the loss of TbPRMT1 protein was not due to off-target RNAi effects as TbPRMT1 RNA levels remained unchanged upon TbPRMT3 repression (Fig.5B). As expected, TbPRMT3 RNA was reduced to levels 24% of those in TbPRMT3-expressing cells (Fig.5B), and TbPRMT6 RNA levels remained unchanged. From these data, together with those presented in Figures1 and 3, we conclude that expression of TbPRMT1 and TbPRMT3 proteins is mutually dependent.

**Figure 6:**
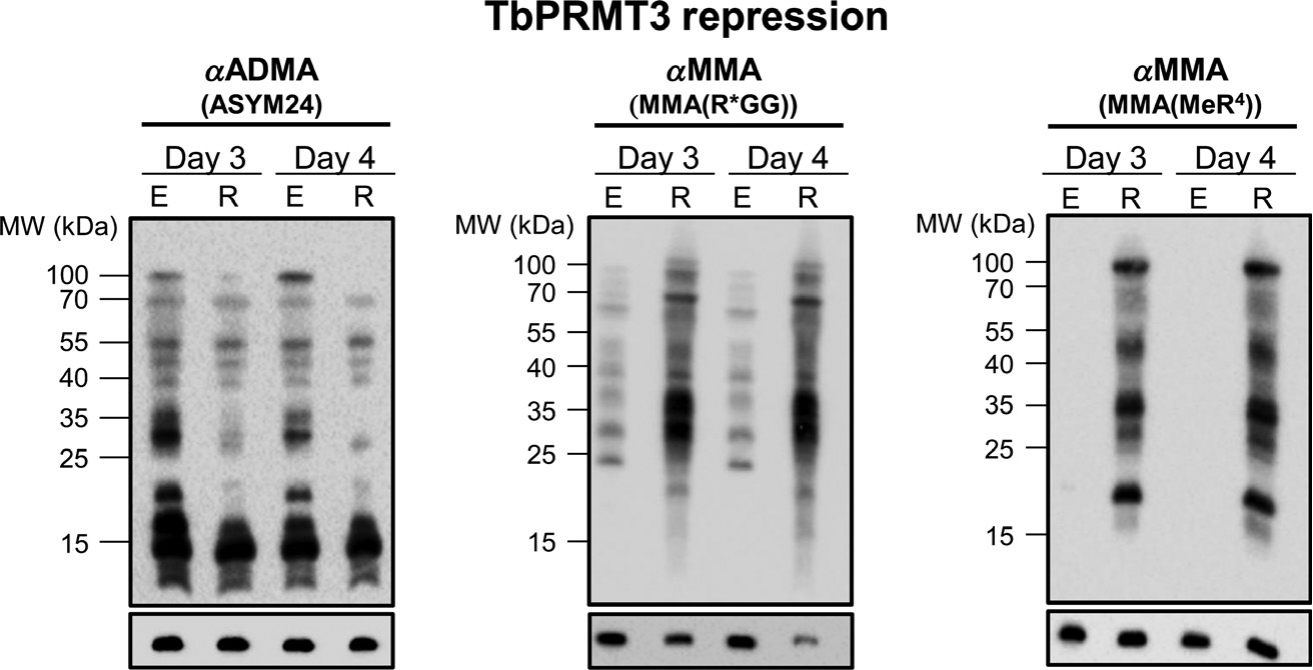
Cells repressed for TbPRMT3 display the same methyl landscape as cells repressed for TbPRMT1. *Trypanosoma brucei* procyclic cells either expressing (E) or repressed (R) for TbPRMT3 were lysed in SDS-PAGE sample buffer and resolved via 12.5% SDS-PAGE. Each lane contains 1 × 10^7^ cell equivalents. Western blot analysis was carried out using three different antimethylarginine antibodies: ASYM24 detects ADMA, and MMA(R*GG) and MMA(MeR^4^) detect MMA. The p22 load control is shown under each blot.

Having shown that TbPRMT3 knockdown results in a similar overall TbPRMT profile as observed in TbPRMT1 knockdown cells (Fig.1), we predicted that the cellular methylation status in TbPRMT3 knockdown cells would be comparable to that seen in the TbPRMT1 knockdown cells. Western blot analysis showed that ADMA levels as detected by ASYM24 indeed exhibited a dramatic decrease similar to that observed in TbPRMT1 knockdown cells (compare Figs.2, 6). There appears to be no additional ADMA loss compared to that observed in TbPRMT1 depleted cells. We next monitored MMA levels in the TbPRMT3 RNAi cells. MMA in both the R*GG and MeR^4^ contexts dramatically increased upon PRMT3 depletion (Fig.6). The MMA pattern that emerged from TbPRMT3 depletion mimics that seen in the TbPRMT1 depletion, with no addition or loss of specific MMA signals. Given that the expression of TbPRMT1 and TbPRMT3 is mutually dependent and knockdown of either enzyme produces similar phenotypes, our data suggest that these enzymes form a functional hetero-oligomer in *T. brucei*, although further studies are needed to define this interaction.

### Simultaneous TbPRMT6/7 knockdown leads to a synergistic effect on parasite growth and suggests TbPRMT6 substrate specificity

**Figure 7:**
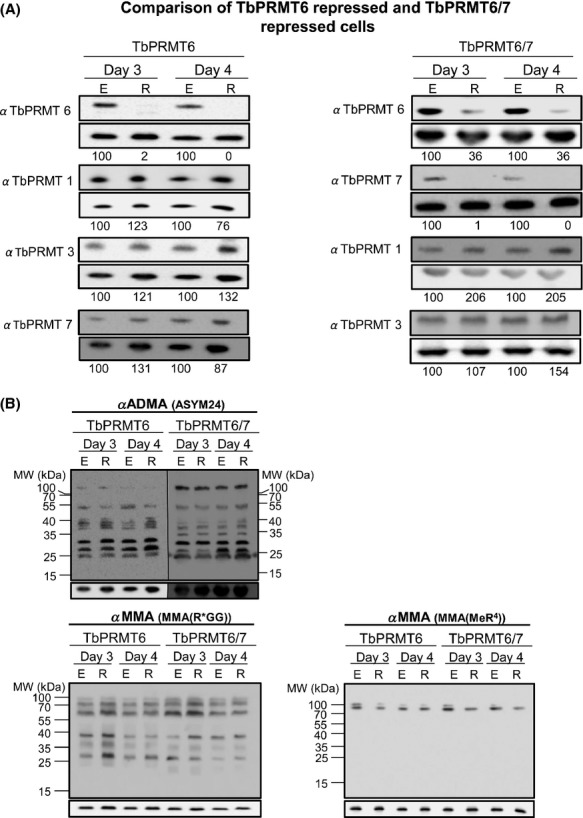
TbPRMT7 does not appear to scavenge TbPRMT6 substrates. (A) *Trypanosoma brucei* procyclic-form cells either expressing (E) or repressed (R) for TbPRMT6 or both TbPRMT6 and TbPRMT7 (TbPRMT6/7) were lysed in SDS-PAGE sample buffer and resolved via 12.5% SDS-PAGE. Each lane contains 5 × 10^6^ cell equivalents. Western blot analysis was carried out using antibodies specific to each TbPRMT. p22 serves as the load control and is shown below each respective western blot. Quantification of bands is represented as normalized percentages compared to PRMT-expressing cells. (B) Western blot analysis was carried out on cell lines from above using three different antimethyl antibodies: ASYM24 detects ADMA, and MMA(R*GG) and MMA(MeR^4^) detect MMA. The p22 is a load control shown under each blot.

We showed above that the type III enzyme, TbPRMT7, methylates substrates that are rendered hypomethylated by repression of the type I TbPRMT1. To determine whether knockdown of the type I TbPRMT6 similarly unmasks TbPRMT7 substrates, we generated cell lines depleted for TbPRMT6 or simultaneously depleted of TbPRMT6 and TbPRMT7. Western blot analysis (Fig.7A) showed that the target proteins were significantly repressed upon tetracycline addition to the culture media. In the TbPRMT6 single knockdown line, TbPRMT6 was depleted to 2% and 0% of wild-type levels at 3 and 4 days postinduction, respectively. In the double knockdown line, TbPRMT6 was depleted to 36% and TbPRMT7 to 1% of wild-type levels. Repression of TbPRMT6 alone or in combination with TbPRMT7 repression caused no reproducible impact on the levels of the TbPRMT1 or TbPRMT3 (Fig.7A).

We previously reported that TbPRMT6 repression leads to a modest slow-growth phenotype in PF and bloodstream form (BF) cells beginning around day 6 postinduction of RNAi, and in vitro methylation assays suggested that TbPRMT6 has a narrow substrate range compared to other TbPRMTs (Fisk et al. 2010). To assess global alterations in the methyl landscape of PF *T. brucei* upon TbPRMT6 depletion, we analyzed TbPRMT6 knockdown cells by western blot analysis. We first analyzed the impact of TbPRMT6 or TbPRMT6/7 knockdown on global ADMA levels as detected with the ASYM24 antibody (Fig.7B). We observed no reproducible decrease in ADMA using this method. The lack of apparent changes in ADMA levels following TbPRMT6 depletion likely reflects both the enzyme's presumably narrow substrate range as suggested by in vitro methylation assays, and the limited view of the total cellular methylome revealed by the ASYM24 antibody due to its context bias (Fisk et al. 2013; Lott et al. 2013). We next asked whether any MMA-containing substrates became unmasked upon TbPRMT6 depletion, as we previously observed in TbPRMT1 depleted cells. Despite observing some variability in the MMA(R*GG) and MMA(MeR4) signals on different days and in the single or double knockdowns, we did not identify any consistent changes in MMA levels in the TbPRMT6 or TbPRMT6/7 cell lines with the two antibodies used (Fig.7B). The apparent absence of any substrate scavenging upon TbPRMT6 repression is consistent with the view that TbPRMT6 methylates a specific and relatively narrow set of substrates.

**Figure 8:**
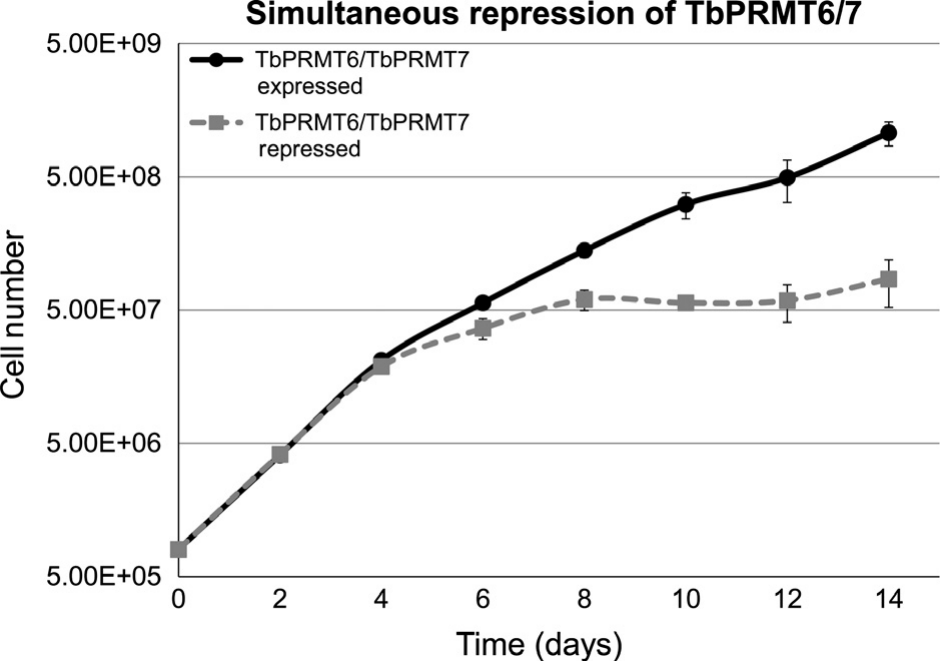
Simultaneous depletion of TbPRMT6 and TbPRMT7 impairs parasite growth. *Trypanosoma brucei* procyclic-form cells either expressing (solid black line) or repressed for (dotted grey line) TbPRMT6 and TbPRMT7 were assessed for parasite growth over the course of 14 days. Cells from triplicate cultures were counted every 2 days and diluted back to a starting concentration of 8 × 10^5^ cells/mL. Error bars represent standard deviation from triplicate experiments.

We next analyzed growth of PF cells simultaneously depleted for TbPRMT6/7 (Fig.8). While these cells grew normally for the first 4 days following tetracycline addition, growth of TbPRMT6/7 repressed cells slowed by day 6 and plateaued by day 8. It is not uncommon for knockdowns of even essential proteins in *T. brucei* to take 6 days to manifest a growth defect (Ammerman et al. 2013), and the time frame for this effect likely depends on a combination of the degree of knockdown at the mRNA level, the stability of the target protein, and the functional importance of the protein. TbPRMT7 PF single knockdowns do not display a growth defect (Fisk et al. 2009), while growth of PF *T. brucei* is modestly affected by TbPRMT6 PF knockdown (Fisk et al. 2010). However, the defect observed in the TbPRMT6/7 repressed cells is much more dramatic than that observed in TbPRMT6 PF single knockdowns under the same conditions (Fisk et al. 2010). Indeed, simultaneous knockdown of TbPRMT6 and TbPRMT7 leads a growth defect reminiscent of that observed when TbPRMT6 repressed cells are subjected to nutrient stress (Fisk et al. 2010). These experiments indicate that wild-type levels and patterns of ADMA and MMA are critical to PF *T. brucei* proliferation.

### TbPRMT7 knockdown suggests the presence of noncanonical type III PRMTs

**Figure 9:**
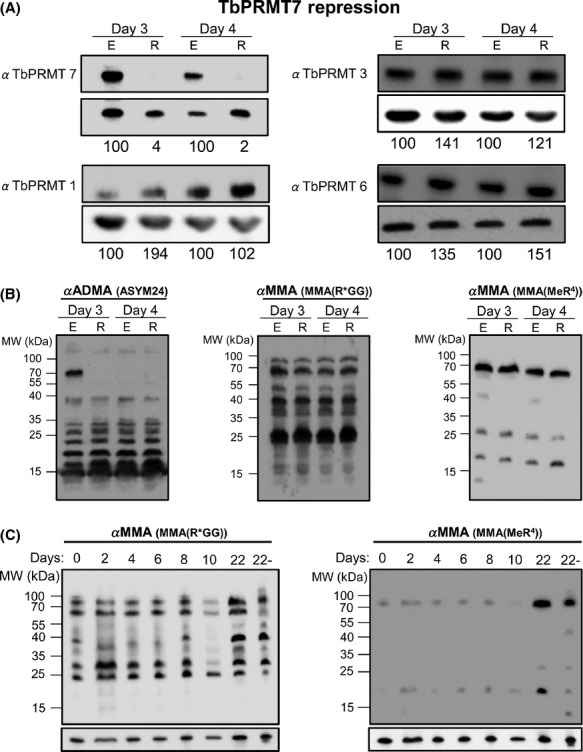
TbPRMT7 repression does not detectably alter MMA levels. (A) *Trypanosoma brucei* procyclic-form cells either expressing (E) or repressed (R) for TbPRMT7 were lysed in SDS-PAGE sample buffer and resolved via 12.5% SDS-PAGE. Each lane contains 5 × 10^6^ cell equivalents. Western blot analysis was carried out using antibodies specific to each PRMT. The p22 load control is shown below each blot. Quantification of bands is represented as normalized percentages compared to PRMT-expressing cells. (B) Western blot analysis was carried out on cell lines shown in 9A using three different antimethylarginine antibodies: ASYM24 detects ADMA, and MMA(R*GG) and MMA(MeR^4^) detect MMA. (C) Western blot analysis of steady-state MMA levels over a 22-day time course following TbPRMT7 repression. The last lane in each blot (22-) indicates cells grown under the same conditions for 22 days in the absence of tetracycline addition. The p22 load control is shown under each blot.

Finally, we wanted to determine the effects of TbPRMT7 repression on the levels of other TbPRMTs and on global arginine methylprotein status. TbPRMT7 is the only identifiable type III PRMT in *T. brucei*, and in vitro it is an exceptionally active enzyme that exhibits robust activity toward a wide range of substrates (Fisk et al. 2009). We demonstrated above that TbPRMT7 acts on numerous substrates that are hypomethylated following TbPRMT1 depletion. In theory, TbPRMT7 could also functionally interact with other PRMTs by synthesizing the MMA intermediate that then acts as a substrate for dimethylarginine (DMA) production by type I and type II PRMTs. To continue to probe the interaction of TbPRMT7 with other TbPRMTs, we analyzed tetracycline-inducible TbPRMT7 knockdowns by western blot to determine the levels of the other PRMTs (Fig.9A). TbPRMT7 expression was decreased to levels nearly below the level of detection upon knockdown, and this did not reproducibly affect the expression of TbPRMT1, TbPRMT3, or TbPRMT6. Thus, PF *T. brucei* cells do not compensate for the loss of TbPRMT7 by changing the expression levels of other TbPRMTs.

Next, we sought to determine the effects of TbPRMT7 repression on the cellular methyl landscape. Because TbPRMT7 is the only apparent type III PRMT in *T. brucei*, we expected that upon its depletion we would see a marked decrease in MMA signal. Moreover, if TbPRMT7 is responsible for catalyzing MMA substrates utilized by other PRMTs for subsequent dimethylation, then we would expect to see a decrease in some ADMA-containing proteins in the absence of TbPRMT7 as well. When we probed total cell lysates of TbPRMT7-expressing or -repressed cells, we observed little significant reduction in ADMA as detected by the ASYM24 antibody, with the exception of a 70-kDa protein whose ADMA mark is compromised upon TbPRMT7 knockdown (Fig.9B, left). Surprisingly, western blots using either MMA(R*GG) or MMA(MeR^4^) antibodies demonstrated that the levels of MMA detectable by these antibodies were also virtually unchanged upon 3 or 4 days repression of TbPRMT7 (Fig.9B, middle and right). Because protein arginine methylation can impact protein stability (Bedford and Clarke 2009; Lee and Stallcup 2009), we considered that monomethylated proteins might be especially stable, and that the MMA signals detected in TbPRMT7 repressed cells might be due to proteins that were methylated prior to TbPRMT7 knockdown and that remained stable over the ensuing 4 days. To test this possibility, we grew TbPRMT7 RNAi cells in the absence or presence of tetracycline for 22 days and analyzed MMA-containing proteins by western blot with MMA(R*GG) or MMA(MeR^4^) antibodies (Fig.9C). Even through this extended time frame, during which TbPRMT7 remained undetectable in tetracycline-induced cells (data not shown), we observed no reproducible diminishment in MMA signal. One possible explanation of this result is that residual, albeit undetectable, TbPRMT7 is capable of fully methylating these substrates. However, we note that a similar degree of TbPRMT7 knockdown leads to dramatically decreased MMA levels in TbPRMT1/7 depleted cells compared to depletion of TbPRMT1 alone (Fig.4). Thus, while it is formally possible that PF *T. brucei* produces a vast overabundance of TbPRMT7 such that very low levels remaining after RNAi are capable of fully methylating some TbPRMT7 substrates, our data are more consistent with the presence of a noncanonical type III PRMT(s) that acts redundantly with TbPRMT7 to produce monomethylated proteins.

## Discussion

In this manuscript, we report the impacts of repressing specific TbPRMTs both on the levels of other TbPRMTs and the global cellular methyl landscape. Our study reveals interplay between different TbPRMTs at numerous levels including those of substrate scavenging, physical interdependence, and synergistic effects on trypanosome growth. In addition, they support our previous conclusion that TbPRMT6 displays a specific and relatively narrow substrate range. Our results also suggest the existence of as-yet-unidentified noncanonical PRMTs in *T. brucei*.

### Potential mechanisms and functions of substrate scavenging

This study demonstrates that TbPRMTs display a functional interplay that can drastically change the cellular methyl landscape. We show here that repression of TbPRMT1 leads to a decrease in ADMA and a substantial increase in MMA decoration of proteins that is catalyzed by TbPRMT7. These results suggest that TbPRMT1 and TbPRMT7 can compete for specific arginine residues. We recently interrogated the methylproteome of *T. brucei* and identified 844 arginine methylated proteins (Fisk et al. 2013; Lott et al. 2013). For 50 of these proteins, we identified distinct peptides in which the same arginine residue contained either MMA or DMA. For example, this class included three RNA helicases (Tb927.5.4420, Tb927.10.14550, and Tb927.9.12510) and a pentratricopeptide repeat protein (Tb927.10.810), all likely involved in RNA processing and/or translation. We also identified several proteins in which two closely spaced modified arginines were present in three to four differentially methylated states with different combinations of MMA and DMA. Interestingly, this class of protein includes two putative tRNA methyltransferases, Tb927.10.9020 and Tb11.01.4510. There are two possible mechanisms by which one arginine can acquire a dimethyl versus a monomethyl mark. First, it is possible that the MMA-containing peptides were recovered from intermediate products prior to being catalyzed to their final DMA states. However, we find it unlikely that this mechanism is in effect in all cases as MMA residues were very abundant in our methylome studies, accounting for approximately half of all identified methylarginines. Another explanation is that arginine methylation is a regulated process, and differentially modified peptides represent proteins with different fates. For instance, TbPRMT1 access to specific substrates could be negatively regulated in response to specific external or developmental cues, thereby favoring the sole access of TbPRMT7 and subsequent monomethylation. Numerous mechanisms for regulation of mammalian PRMTs have been reported (Pahlich et al. 2006), including both protein–protein interactions and phosphorylation, and it is likely that similar regulatory processes are at work in *T. brucei*. Recent phosphoproteome studies in *T. brucei* identified four sites of phosphorylation in TbPRMT3 (Urbaniak et al. 2013), and proteomic studies in progress in our laboratory are likely to reveal TbPRMT interaction partners, including regulatory molecules. Modulation of TbPRMT1 and TbPRMT7 access to the same substrate is likely to have dramatic consequences for protein function, similar to what has been observed in other systems. For example, ADMA on mammalian histone H3 R2 is linked to transcriptional repression, while MMA on the same residue is associated with gene activation (Schurter et al. 2001; Guccione et al. 2007; Migliori et al. 2012). The mechanisms by which arginine methylation is regulated and the functional distinctions between differentially modified proteins will be important for a full understanding of protein arginine methylation in parasite biology and development.

### TbPRMT1 and TbPRMT3 exhibit interdependence

When analyzing cells repressed for specific TbPRMTs, we unexpectedly observed a strict interdependence between TbPRMT1 and TbPRMT3 protein levels (Figs.1, 5). The simplest explanation of this finding is that when one of these proteins is absent, the other becomes unstable and is subsequently degraded. As this type of behavior is commonly seen in protein complexes, our data suggest that TbPRMT1 and TbPRMT3 form a hetero-oligiomeric complex in vivo. Interestingly, mammalian PRMT3 was first identified through a yeast-two-hybrid screen using Rat PRMT1 as bait, although further attempts at recovering a physiological PRMT1/PRMT3 complex were unsuccessful (Tang et al. 1998). In addition, these two TbPRMTs were among the small number of proteins recently coprecipitated with the arginine methylated *T. brucei* protein, SCD6 (Cristodero et al. 2014). PRMTs generally function as homodimers (Zhang et al. 2000; Zhang and Cheng 2003; Cheng et al. 2011; Sun et al. 2011). However, there are reports of heterodimer formation, particularly with respect to PRMT1. For example, human PRMT8 can dimerize with human PRMT1 when exogenously expressed in cultured HeLa or HEK293 cells (Lee et al. 2005; Dhar et al. 2013). However, PRMT8 expression is restricted to the central nervous system (Lee et al. 2005; Kousaka et al. 2009), and to our knowledge there is no evidence of a native PRMT1/PRMT8 heterodimer. Mammalian PRMT1 and PRMT2 can also interact when expressed in cultured cells and when recombinant proteins are mixed in vitro (Pak et al. 2011). Interestingly, in vitro methylation assays showed that either PRMT2 or an inactive PRMT2 mutant stimulated PRMT1 activity by ∼sixfold, by increasing both the Vmax and kcat of PRMT1. This raises the intriguing possibility that mammalian PRMT1 may heterodimerize with other PRMTs that, in turn, modulate its activity. Whether such physical and functional interactions occur between endogenous mammalian PRMTs awaits further study.

In our hands, the enzymatic activity of TbPRMT1 is exceedingly low, and despite significant efforts we have never been able to detect activity of TbPRMT3 in vitro. We show here, however, that depletion of these enzymes in vivo results in a significant effect on global ADMA levels. These findings, together with the physical codependence of TbPRMT1 and TbPRMT3, suggest that TbPRMT1 and TbPRMT3 form a functional complex. This scenario is reminiscent of the prozyme–enzyme complexes described in *T. brucei* by the Phillips group (Willert et al. 2007; Nguyen et al. 2013). In this scenario, catalytically dead homologues of an active enzyme stimulate the enzyme's activity upon binding. For example, the AdoMetDC/prozyme complex catalyzes an important step in polyamine biosynthesis (Willert and Phillips 2012). The prozyme arose from a gene duplication of AdoMetDC followed by mutations of key catalytic residues, and as a result the enzyme and prozyme share ∼30% sequence identity (Willert et al. 2007). Much like AdoMetDC and its prozyme, TbPRMT1 and TbPRMT3 are 27% identical, and TbPRMT3 is also lacking several key catalytic residues that are typically conserved in type I PRMTs. Upon binding of the prozyme, AdoMetDC activity is stimulated by 1200-fold. The other reported prozyme-activated enzyme is deoxyhypusine synthase (Nguyen et al. 2013). Interestingly, repression of either deoxyhypusine synthase or its prozyme leads to simultaneous degradation of both proteins, similar to our observations with TbPRMT1 and TbPRMT3. Thus, it is tempting to speculate that TbPRMT3 is an enzymatically dead PRMT homologue that interacts with TbPRMT1 to stimulate TbPRMT1 activity in vivo. Future experiments will be aimed at testing this model.

### TbPRMT7, MMA, and Noncanonical PRMTs

TbPRMT7 is a *bone fide* type III PRMT, solely catalyzing the production of MMA residues, and it is the only canonical type III PRMT apparent in the *T. brucei* genome. In vitro it displays a high degree of activity and a broad substrate range (Fisk et al. 2009). Therefore, we expected that repression of TbPRMT7 would result in a dramatic loss of MMA. On the contrary, we found that the MMA signals identified by either of two anti-MMA antibodies did not decrease upon TbPRMT7 depletion, even when TbPRMT7 was repressed for 22 days (Fig.9). One possible explanation is that the residual amount of TbPRMT7 that remains after knockdown (2% and 0% on days 3 and 4 postinduction, respectively) is sufficient for catalysis of wild-type MMA levels. Contradicting this model, however, is our finding that a similar degree of TbPRMT7 repression was sufficient to dramatically decrease MMA production in the TbPRMT1 knockdown background (Fig.4). These two conditions do differ with respect to the available TbPRMT7 substrates. When TbPRMT1 is repressed, numerous substrates normally dimethylated by TbPRMT1 are unmasked and made available to TbPRMT7. It is possible that TbPRMT7 has a much lower affinity for the unmasked substrates than it does for its primary targets in TbPRMT1-replete cells. If this is true, decreasing the cellular TbPRMT7 concentration may affect methylation of the lower affinity unmasked pool of substrates, while other substrates for which TbPRMT7 has high affinity can still be methylated by the remaining low levels of the enzyme. Recently, it was reported that the preferred motif of murine PRMT7 is a stretch of basic amino acids containing at least two arginines in close proximity (Feng et al. 2013). Therefore, it is possible that the discrepancy we observe in the MMA signals upon TbPRMT7 knockdown in the presence and absence of TbPRMT1 could be due to the motifs surrounding the target arginines in the different substrate pools.

Another possible explanation of why TbPRMT7 repression does not cause an apparent decrease in the global MMA signal could be the presence of noncanonical PRMTs that act in a redundant fashion with TbPRMT7. The existence of noncanonical TbPRMTs was also suggested by a previous study, in which we identified 168 arginine methylated proteins in *T. brucei* mitochondria (Fisk et al. 2013) despite our inability to detect any of the characterized TbPRMTs in this subcellular location. A search of the annotated *T. brucei* database (TriTrypDB.org) reveals that *T. brucei* encodes 117 genes with a predicted methyltransferase domain. While many of these undoubtedly methylate nonprotein substrates, there have been recent reports from other organisms characterizing novel PRMTs. Human NDUFAF7, a mitochondrial membrane protein involved in mitochondrial complex I formation, was shown to symmetrically methylate the NDUFS2 subunit of complex 1 early in complex formation (Rhein et al. 2013). *T. brucei* encodes a putative homologue of human NDUFAF7, which is predicted to localize to the mitochondria. Another reported noncanonical PRMT is the *S. cerevisiae* protein, Yor021c, which belongs to the family of SPOUT methyltransferases (Petrossian and Clarke 2009). SPOUT methyltransferases were first identified based on their common structural fold that is distinct from the Rossmann fold found in other methyltransferases (Anantharaman et al. 2002; Tkaczuk et al. 2007), and several members of this family methylate both tRNA and rRNA (Sirum-Connolly and Mason 1993; Cavaille et al. 1999; Jackman et al. 2003; Meyer et al. 2011). Recently, Yor021c was shown to catalyze the formation of MMA on Arg146 in the ribosomal protein Rps3 (Young et al. 2012). A search of the *T. brucei* database revealed that *T. brucei* contains five SpoU domain-containing proteins, which we now must consider as potential PRMTs. Characterizing new methyltransferases and determining enzyme/substrate pairs will greatly expand our understanding of the biological roles of protein arginine methylation.

*Trypanosoma brucei* has a complex life cycle with two hosts and alternative dividing and nondividing forms, necessitating developmental regulation to adapt to large environmental changes. Uniquely, *T. brucei* gene regulation is primarily posttranscriptional, relying on RNA-binding proteins to regulate cell fate (Clayton and Shapira 2007). Interestingly, a recent proteomics screen identified many arginine methylproteins involved in RNA metabolism, suggesting that TbPRMTs, and the methylation which they catalyze, may have a significant impact on parasite development (Lott et al. 2013). In higher eukaryotes, PRMTs can regulate cell development and differentiation. For example, in *Drosophila*, arginine methylation by their PRMTs (DARTS) has been linked to establishment of germ cells and wing development (Anne 2010; Xu et al. 2013). Recently, expression of human PRMT4 was shown to regulate myeloid differentiation of human stem cells, thus making PRMT4 an attractive chemotherapeutic target for myelogenous leukemia (Vu et al. 2013). Finally, the field of epigenetics has uncovered several mechanisms by which PRMTs decide cell fate (Di Lorenzo and Bedford 2011). Therefore, regulation of TbPRMTs may be one likely way in which methylation affects developmental changes in *T. brucei*.
